# Do aphids in Dutch sweet pepper greenhouses carry heritable elements that protect them against biocontrol parasitoids?

**DOI:** 10.1111/eva.13347

**Published:** 2022-02-15

**Authors:** Mariska M. Beekman, Suzanne H. Donner, Jordy J. H. Litjens, Marcel Dicke, Bas J. Zwaan, Eveline C. Verhulst, Bart A. Pannebakker

**Affiliations:** ^1^ Laboratory of Genetics Wageningen University & Research Wageningen The Netherlands; ^2^ Laboratory of Entomology Wageningen University & Research Wageningen The Netherlands

**Keywords:** *Aphidius* parasitoids, *Aulacorthum solani*, biocontrol, defensive symbiosis, endogenous resistance, *Myzus persicae*

## Abstract

Biological control (biocontrol) of crop pests is a sustainable alternative to the use of biodiversity and organismal health‐harming chemical pesticides. Aphids can be biologically controlled with parasitoid wasps; however, variable results of parasitoid‐based aphid biocontrol in greenhouses are reported. Aphids may display genetically encoded (endogenous) defences that increase aphid resistance against parasitoids as under high parasitoid pressure there will be selection for parasitoid‐resistant aphids, potentially affecting the success of parasitoid‐based aphid biocontrol in greenhouses. Additionally, aphids may carry secondary bacterial endosymbionts that protect them against parasitoids. We studied whether there is variation in either of these heritable elements in aphids in greenhouses of sweet pepper, an agro‐economically important crop in the Netherlands that is prone to aphid pests and where pest management heavily relies on biocontrol. We sampled aphid populations in organic (biocontrol only) and conventional (biocontrol and pesticides) sweet pepper greenhouses in the Netherlands during the 2019 crop growth season. We assessed the aphid microbiome through both diagnostic PCR and 16S rRNA sequencing and did not detect any secondary endosymbionts in the two most encountered aphid species, *Myzus persicae* and *Aulacorthum solani*. We also compared multiple aphid lines collected from different greenhouses for variation in levels of endogenous‐based resistance against the parasitoids commonly used as biocontrol agents. We found no differences in the levels of endogenous‐based resistance between different aphid lines. This study does not support the hypothesis that protective endosymbionts or the presence of endogenous resistant aphid lines affects the success of parasitoid‐based biocontrol of aphids in Dutch greenhouses. Future investigations will need to address what is causing the variable successes of aphid biocontrol and what (biological and management‐related) lessons can be learned for aphid control in other crops, and biocontrol in general.

## 1 INTRODUCTION

Aphids are among the most damaging pests for greenhouse crops (van Emden & Harrington, 2017). Notably, the salivary excretion of many aphid species is phytotoxic, causing stunting, galls and leaf malformations. Furthermore, aphids transmit numerous agriculturally important plant viruses (Katis et al., 2007; Nault, 1997). Aphids can reproduce through parthenogenesis and regularly develop high population densities in greenhouses as these offer favourable environments to aphids in terms of temperature and food sources. Therefore, there is an urgent need for effective control of greenhouse aphid populations.

In the past, aphid outbreaks have been controlled mainly with pesticides. However, many chemical pesticides are not target‐specific and are toxic for a wide variety of organisms (Ansari et al., 2014), and legislation now restricts their use (European Parliament, 2009, 2013). In contrast, biological control (biocontrol), which uses natural enemies to reduce pest populations, is a more sustainable pest control method. Many natural enemies suitable for biocontrol are available, but parasitoid wasps are considered especially useful because of their short generation time, high fecundity and rapid dispersal throughout the crop, and because the choice of parasitoid species allows for exclusively targeting aphids (Rabasse & van Steenis, 1999).

As discussed by other researchers, successful biocontrol of aphids in greenhouse crops can be a challenge (Boivin et al., 2012; Glastuinbouw Nederland, 2020; Messelink et al., 2014; Sanchez et al., 2007) and there are many management‐related and biological factors that might explain the variable success of parasitoid‐based aphid biocontrol. Examples of management‐related factors are the quantity and frequency of parasitoid release (Hopper & Roush, 1993), provision of shelter, food and hosts to promote preventative establishment of natural enemies in the crop (standing army; Messelink et al., 2014; Pijnakker et al., 2020), and the usage of pesticides with untargeted side effects on biocontrol agents (Alfaro‐Tapia et al., 2021; Cloyd & Bethke, 2011). Altogether, the management of aphid pest control is highly affected by the type of farming management practised in a greenhouse, whether it be organic or conventional. Organic greenhouses exclude all input from chemical sources and rely solely on natural enemies for aphid control. Conventional greenhouses that make use of parasitoids for aphid control combine this with the occasional usage of pesticides to quickly correct massive pest population growth. A potentially important, but currently underexplored, biological factor that may affect the success of parasitoid‐based control of aphids in greenhouses is the role of heritable elements involved in parasitoid resistance. Heritable elements that can protect aphids against parasitoid wasps can be secondary bacterial endosymbionts (Vorburger, 2018), or host genes providing so‐called endogenous resistance (Martinez et al., 2014, 2016, 2018; Sandrock et al., 2010).

The most‐studied protective aphid endosymbiont is the bacterium *Hamiltonella defensa* (Moran et al., 2005). It protects aphids against endoparasitoid wasps when it carries a bacteriophage called *Acyrthosiphon pisum* secondary endosymbiont (APSE; Weldon et al., 2013). Several APSE strains encode different toxin analogues that are hypothesized to kill the developing wasp larvae (Brandt et al., 2017; Degnan & Moran, 2008). Apart from *H*. *defensa*, specific strains of other bacterial endosymbionts, such as *Regiella insecticola* (Moran et al., 2005), *Fukatsuia symbiotica* (Manzano‐Marín et al., 2017) and *Spiroplasma* sp., can also protect aphids against parasitoids (Heyworth & Ferrari, 2015; McLean et al., 2020; von Burg et al., 2008). Important to note is that in all these cases the protective effects of endosymbionts are specific to the species or even the genotype of the endosymbiont, aphid and/or parasitoid (Hopper et al., 2018; McLean & Godfray, 2015; McLean et al., 2020; Oliver & Higashi, 2019; Rouchet & Vorburger, 2012), making the protective effects of endosymbionts highly context‐dependent.

Many laboratory and field studies have shown that protective endosymbionts can strongly affect aphid–parasitoid population structure and dynamics, and can even lead to parasitoid extinction (Käch et al., 2018; Oliver et al., 2008; Rothacher et al., 2016; Sanders et al., 2016). However, in greenhouses that rely on parasitoids for aphid control, the actual presence of endosymbionts and potential effects on aphid–parasitoid dynamics have rarely been studied. These greenhouses frequently deploy a substantial number of parasitoid wasps, a pest management strategy known as augmentative biocontrol. It is hypothesized that, especially in organic greenhouses, this will lead to a high parasitoid‐induced selection pressure that selects for endosymbiont‐protected aphids. Consequently, it could result in rising endosymbiont frequencies in greenhouse aphid populations as has been observed in laboratory cage studies (Vorburger, 2018), which could result in difficulties for aphid biocontrol. In particular, when aphid populations can remain in a greenhouse system during subsequent years, for example by hiding on weeds present in the greenhouse, or in natural habitats surrounding the greenhouse, evolution of parasitoid‐resistant aphid populations could occur. We expect that protective endosymbionts are less of a problem in conventional greenhouses as parasitoid‐resistant aphids can still be killed by pesticides.

A previous study that focused on the effects of endosymbionts on aphid–parasitoid dynamics in a greenhouse crop was recently published by Postic et al. (2020). They showed that in French greenhouse strawberry crops, one of the most common aphids on strawberries, *Acyrthosiphon malvae rogersii* (Theobald), often carried *H*. *defensa* or a combination of *H*. *defensa* and *R*. *insecticola*. The parasitism success of the parasitoid *Aphidius ervi* Haliday on these symbiont‐infected aphids was significantly reduced (from 37.7% to 1.6% and 0.2%, respectively) compared with uninfected aphids of the same species. This study shows that endosymbionts may affect biocontrol success of greenhouse aphids and warrants studies in other countries and in other crop systems.

Apart from relying on bacterial endosymbionts for protection against natural enemies, aphids also display intrinsic resistance, also known as endogenous defences or endogenous‐based resistance. The cellular resistance pathways of aphids against parasitoids are still mostly unknown. Principal elements of signalling pathways in other insects known to be involved in immunity against parasitoids, microbes and other stresses are missing from the aphid genome, indicating that the endogenous resistance mechanisms of aphids are different from those of other insects (Gerardo et al., 2010). Multiple studies have shown that endosymbiont‐free lines of the pea aphid *Acyrthosiphon pisum* (Harris) have highly variable resistance levels against the parasitoid *A*. *ervi* (Doremus et al., 2018; Martinez et al., 2014, 2016; McLean & Parker, 2020). Also in the black bean aphid, *Aphis fabae* Scolopi (Sandrock et al., 2010) and the peach‐potato aphid *Myzus persicae* (Sulzer), significant clonal variation in resistance against parasitoids has been observed (von Burg et al., 2008). It can be expected that other aphid species also display variable levels of endogenous‐based resistance against parasitoids. Even for aphids carrying protective endosymbionts, endogenous‐based resistance mechanisms may still be important, as endosymbiont‐based resistance may fail under specific conditions such as elevated temperatures (Martinez et al., 2018).

Thus, endosymbiont‐based resistant aphids are suggested to affect the success of biocontrol in greenhouse systems (Vorburger, 2018) and aphids display variable levels of endogenous‐based resistance against parasitoids in laboratory studies (Doremus et al., 2018; Martinez et al., 2014, 2016; McLean & Parker, 2020; Sandrock et al., 2010; von Burg et al., 2008). Yet, studies on the presence and effects of parasitoid‐resistant aphids, either caused by endosymbionts or encoded endogenously, in greenhouses using biocontrol are scarce. A first step to study whether such protective heritable elements affect biocontrol of greenhouse aphids is to study the presence of such heritable elements in greenhouse aphids. Consequently, the aim of this study was to determine whether secondary endosymbionts and/or variation in levels of endogenous resistance against parasitoids are present in greenhouse aphids. To study this, we focused on the crop sweet pepper, *Capsicum annuum L*. (Solanaceae), grown in greenhouses in the Netherlands. Sweet pepper is an agro‐economically important crop in the Netherlands (Breukers et al., 2008) that is prone to highly polyphagous aphid pests (Blackman & Eastop, 2000; Messelink et al., 2011; Rabasse & van Steenis, 1999) and heavily relies on parasitoid‐based biocontrol of these aphids, both in organic and conventionally managed greenhouses (CBS, 2021). Altogether, this makes sweet pepper in the Netherlands a highly relevant study system to investigate the presence of endosymbionts and endogenous‐based resistance in aphids with high economic importance, under high (organic greenhouses using biocontrol only) and low (conventional greenhouses using biocontrol and pesticides) selection pressure by parasitoids.

In this study, we sampled aphids from organic and conventional sweet pepper greenhouses located in the Netherlands during the crop growth season of 2019. Our objectives were as follows: (1) to gain insight into the most common aphid lines (aphid species/endosymbiont combinations) present throughout greenhouses and to follow the dispersal of these aphid lines throughout the greenhouse over the crop growth season through aphid sampling. Additionally, we aimed to gain insight into the management of aphid control, in both organic and conventional greenhouses, through interviews with the growers focusing on their usage of biocontrol agents, pesticides and perceived biocontrol success; (2) to assess the presence of secondary aphid endosymbionts through diagnostic PCR and complete microbiome sequencing; and (3) to test for variation in levels of endogenous defences against parasitoids in aphids from different greenhouses through parasitism assays with the common biocontrol agents *A*. *ervi*, *Aphidius colemani* Vierick and *Aphidius matricariae* Haliday.

## 2 MATERIALS AND METHODS

### 2.1 Aphid communities, pest management and biocontrol success as perceived by growers

#### 2.1.1 Greenhouses and aphid collection

**FIGURE 1:**
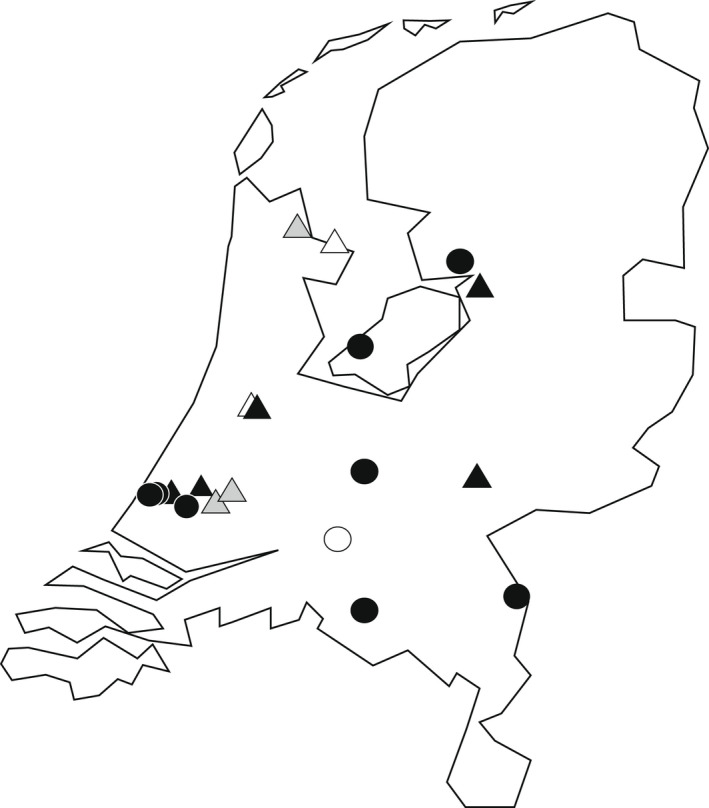
Aphid sampling locations from sweet pepper greenhouses in the Netherlands. Circles represent organic greenhouses; triangles, conventional greenhouses. The shading of the shapes shows whether aphids were collected during one (white), two (grey) or all three (black) of the sampling time points

**FIGURE 2:**
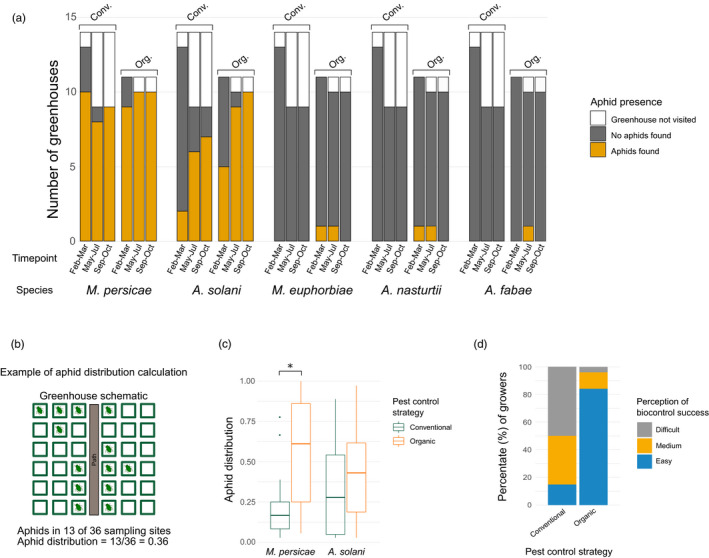
Aphid communities and distribution in organic and conventional sweet pepper greenhouses in the Netherlands. (a) Overview of the aphid species found in sweet pepper greenhouses divided by sampling time point and split for organic (org.) and conventional (conv.) pest control strategies. (b) Example of this distribution calculation for a greenhouse. Aphid distributions were calculated as the proportion of the search grids we found aphids in. (c) Boxplots of the distribution of *Myzus persicae* and *Aulacorthum solani*. *M*. *persicae* had a higher dispersal in organic greenhouses than in conventional greenhouses (GLMM: χ²(1, *N* = 1989) = 22.59, *p* < .001), but *A*. *solani* did not (GLMM: χ²(1, *N* = 1989) = 2.14, *p* = .143). (d) Perceived ease of aphid biocontrol by greenhouse growers. Organic growers find aphid populations easy to control significantly more often than conventional growers (84% vs 15% considered aphid control ‘easy’, respectively, χ²(1, *N* = 45) = 21.25, *p* < .001)

Aphids were sampled in 2019 in sweet pepper crops in greenhouses (enclosed glass buildings used for commercial crop production) in the Netherlands. We sampled 16 growers, having greenhouses at 19 locations, representing a total of 26 greenhouse compartments (Figures 1 and 2a; Table S1). We aimed to obtain an overview of the most abundant aphid lines (aphid species/endosymbiont combinations) present throughout the greenhouse and intended to follow the dispersal of endosymbiont‐infected aphid lines throughout the greenhouse over time. Therefore, most greenhouse compartments were divided into a 6 × 6 grid (Figure 2b), independent of greenhouse size, and we searched for aphids in each cell of the grid (henceforth called ‘sampling sites’). Some greenhouse compartments were notably smaller or of alternative shape, and were divided into an alternative grid (e.g. 3 × 3 and 8 × 3; Table S1). If present, multiple aphids per colony were collected and stored in 70% ethanol at −20°C until further use. Aphids were morphologically identified to the species level using the identification keys of Blackman and Eastop (2000).

Greenhouses were sampled three times, in February/March, May/June/July and September/October, representing the start, middle and end of the crop growth season. In some cases, we did not sample a greenhouse at one of the time points when the grower reported that there were no aphids present, in all but one cases because of recent insecticide treatment (Figure 2a; Table S1), and once because the entire crop was discarded due to nematode infestation. We did not include these unvisited greenhouses in any further analyses.

The regulations that determine whether a greenhouse crop can be considered organic differ between countries. Here, we categorized greenhouse crops as organic when the growers did not use any chemical pesticides. This includes both growers that grow sweet peppers in soil and in suspended substrate systems. Greenhouses that occasionally used chemical pesticides, next to the (periodically) augmentative release of parasitoids, were categorized as conventional.

#### 2.1.2 Interviews with greenhouse growers

To gain an impression of the differences in pest management activities between the greenhouses, we conducted an interview with each grower. First, we asked basic information about the greenhouse itself, such as greenhouse size and crop varieties. Next, we asked the grower to describe in detail which types of pest control were used. This included the name of any chemical pesticides and biocontrol agents, their brand, frequency of use and amounts. Lastly, to check whether the presence of specific aphid lines affects how the grower perceived the success of their biocontrol agents, we asked the growers about their perception of the ease of aphid biocontrol they experienced this year: easy, medium or hard.

### 2.2 Detection of bacterial endosymbionts

#### 2.2.1 DNA extractions and diagnostic PCRs

Aphids were rinsed thoroughly with sterile ultrapure water and mechanically homogenized in 100 μl 5% Chelex 100 resin (Bio‐Rad, Hercules, CA, USA) in ultrapure water with 2.5 μl proteinase K (20 mg/ml, Promega, Southampton, UK). Samples were vortexed, incubated for at least one hour at 56°C, vortexed again and incubated at 96°C for eight minutes. After centrifugation at full speed, the supernatant was transferred to a new tube and diluted 1:2 in ultrapure water.

To reduce the workload of DNA extractions and PCRs, DNA was extracted from single aphids from the four most distant sampling sites in the greenhouse. When more than four aphids of the same species were found in one greenhouse compartment, we extracted the DNA of the rest of the samples in pools of up to 12 aphids. We showed that in these pooled samples of up to 20 aphids, endosymbionts originally present in only a single aphid could be detected (Text S2).

We screened the extracted aphid DNA for the presence of the most common secondary endosymbionts using diagnostic PCR with species‐specific primers for the 16S rRNA gene. We used three multiplex PCR mixes to screen for the secondary endosymbionts *Hamiltonella defensa*, *Regiella insecticola*, *Serratia symbiotica*, *Fukatsuia symbiotica*, *Arsenophonus* sp., *Spiroplasma* sp., *Rickettsiella* sp. and *Rickettsia* sp. Primers to detect *Buchnera aphidicola* (Munson et al., 1991), the primary endosymbiont present in nearly all aphids, were included as a positive control, and only samples that tested positive for *Buchnera* were considered successful and included in the analysis. The performance of all primers was tested beforehand on aphid samples known to contain the respective secondary endosymbionts. All primers, their references, concentrations and the multiplex combinations, can be found in Table S2. Reactions were performed by adding 1 µl of DNA to 9 µl GoTaq^®^‐based PCR Mastermix (Promega), prepared according to the manufacturer's instructions with a final Mg^2+^ concentration of 2.5 mM.

The PCR programme for all multiplexes was as follows: initial denaturation at 94°C for 3 min; 35 cycles of 94°C for 30 s, 58°C for 30 s and 72°C for 60 s; followed by 72°C for 10 min. The success of the PCR was confirmed by gel electrophoresis using a 1% agarose gel, stained with ethidium bromide, with a GeneRuler 100bp DNA Ladder (Thermo Fisher Scientific). Positive multiplex results were confirmed using a singleplex PCR for the detected endosymbiont, and samples were sent for Sanger sequencing to Eurofins Genomics. The resulting sequences were BLASTed against the NCBI nucleotide database (NCBI Resource Coordinators, 2018) to find a species match.

#### 2.2.2 Complete microbiome analysis

To investigate whether any endosymbionts were undetected by diagnostic PCR, we used nanopore sequencing (Oxford Nanopore Technologies, Oxford, UK) of the 16S rRNA gene to profile the complete microbiomes of a subsample of the collected aphids. Per greenhouse compartment, aphid species and sampling time point, one sample consisting of four aphids taken from the outermost sampling sites of the greenhouse was analysed.

The aphids were checked under a stereomicroscope to confirm species identity and to avoid visibly parasitized aphids (bloated/ unusual dark coloration/ wasp larva visible). Next, the aphids were surface‐sterilized (30 s‐5 min) in commercial 4% bleach and rinsed in sterile ultrapure water, after which DNA was extracted using the DNeasy PowerSoil Kit (Qiagen, Venlo, the Netherlands). For this, the aphids were added to 60 μl solution C1, 500uL PowerSoil bead solution and 100 μg proteinase K, after which they were mechanically homogenized in the solution and incubated overnight at 56°C. The following day, the PowerSoil protocol was followed including 10‐ to 20‐min shaking using a vortex. Final DNA was eluted in 30 μl of solution C6. The ZymoBIOMICS Microbial Community Standard (D6300, Zymo Research, Irvine, CA, USA) was included as a positive control. A negative control consisted out of all reagents and extraction steps, without aphids.

The 16S rRNA gene was PCR‐amplified using the primer pair 27F/1492R (Lane, 1991) with the ONT Universal Tags added to the 5’ end, which allowed for attaching barcodes later (see Table S2 for the primer sequences). The reactions were carried out in 25 μl volumes containing 1 μl DNA, 1× Phusion High‐Fidelity buffer, 0.5 μM of each primer, 200 μM dNTPs and 0.02 U/μl Phusion High‐Fidelity polymerase (Thermo Fisher Scientific). The ZymoBIOMICS Microbial Community DNA Standard (D6305) was included as a positive PCR control. The PCR conditions were as follows: initial denaturation at 98°C for 30 s; 25 cycles of 98°C for 10 s, 51°C for 30 s and 72°C for 45 s; followed by 72°C for 5 min.

A clean‐up of the amplicons was performed using a 0.8× volume of homemade SPRI beads (1 ml Sera‐Mag SpeedBeads (Cytiva, Marlborough, MA, USA) cleaned and dissolved in 50 ml end volume containing 2.5 M NaCL, 20 mM PEG, 10mM Tris‐HCL and 1 mM EDTA). Cleaned amplicons were eluted in 15 μl sterile ultrapure water. Concentrations were measured using Qubit^®^ 2.0 fluorometer (Invitrogen) with Qubit^®^ dsDNA HS Assay Kit (Thermo Fisher Scientific), and the volumes were adjusted with sterile ultrapure water to ensure that the barcoding PCR was performed using 0.4–0.8 μM of the 16S PCR product.

To enable the pooling of samples, the 16S PCR products were barcoded using the PCR Barcoding Expansion 1‐96 (Oxford Nanopore Technologies). The reactions were carried out in 15 μl volumes, containing 1× LongAmp® Taq Master Mix (New England Biolabs, Ipswich, MA, USA), 0.4–0.8 μM of 16S PCR product and 0.2 μM barcode. The PCR conditions were as follows: initial denaturation at 95°C for 3 min; 13 cycles of 95°C for 15 s, 62°C for 15 s and 65°C for 80 s; followed by 65°C for 2 min. The concentrations of the resulting amplicons were measured using Qubit^®^ fluorometer, all amplicons were pooled in equimolar concentrations, and the pool was cleaned using the SPRI bead method described above. The final pool of 1000 ng in 47 μl was repaired, end‐prepped and adaptor‐ligated using the NEBNext FFPE DNA Repair Mix (M6630), NEBNext End repair/dA‐tailing Module (E7546) and the NEBNext Quick Ligation Module (E6056) (New England Biolabs) according to the Oxford Nanopore Technologies PCR barcoding (96) amplicons (SQK‐LSK109) protocol, version PBAC96_9069_v109_revM_14Aug2019 (from https://community.nanoporetech.com/docs/prepare/library_prep_protocols).

Sequencing was performed using a R9.4.1 SpotON Flow Cell Mk I (FLO‐MIN106) on a MinION Mk1C sequencing device (Oxford Nanopore Technologies) and ran for 20 h. Basecalling of the nanopore signals, and subsequent demultiplexing, was performed by the embedded MinKNOW software version 19.12.12 through the integrated Guppy software version 3.2.10.

To determine the length of the reads and the number of bases that needed to be trimmed to remove adapters, barcodes and primers, we visually checked the raw reads using Geneious Prime v. 2019.1.3. (BioMatters Ltd., Auckland, New Zealand). After the visual check, files containing the raw reads were merged into a single fastq file per barcode. The reads were filtered using NanoFilt version 2.7.1 (https://github.com/wdecoster/nanofilt) for quality score 10, and lengths between 1500 and 1750 bp. Subsequently, 115 bases from both the beginning and end of the reads were trimmed. Taxonomic assignment was performed with a k‐mer‐based approach using Kraken 2 (Wood et al., 2019), with the Prokaryotic RefSeq Genomes from the NCBI Reference Sequence Database (downloaded on 15 February 2021 from https://www.ncbi.nlm.nih.gov/refseq/about/prokaryotes/) as the reference sequences. Separate Kraken 2 reports were merged into a single file using the tool Kraken2‐output‐manipulation (https://github.com/npbhavya/Kraken2‐output‐manipulation).

After taxonomic assignment, the data set was cleaned by removing spurious taxa to control for sequencing artefacts, sequencing errors and incorrectly assigned barcodes (Cao et al., 2021). This was based on the results of the ZymoBIOMICS microbial DNA, and the ZymoBIOMICS microbial community standard, by discarding any reads assigned to a genus when these reads made up <0.5% of the total reads in a sample (see Text S1 for detailed methods). Subsequently, to enable comparison between samples during downstream analyses, the data were rarefied using the ‘rarefy_even_depth’ function of the R package *phyloseq* v. 1.28.0’ (McMurdie & Holmes, 2013). The final data set was visualized with the R package *microbiome* v. 1.6.0’ (Lahti & Shetty, 2017). R analyses were done in RStudio (v. 1.1.463; RStudio Team, 2016) using R (v. 3.6.1; R Core Team, 2019).

### 2.3 Screening for endogenous resistance

We aimed to test whether variation in endogenous resistance was present in aphid lines from different greenhouses. We expected these aphid lines to display various levels of endogenous resistance to parasitoid wasps, as we hypothesize this to be one of the traits under selection due to high parasitoid pressure, potentially leading to the evolution of parasitoid‐resistant greenhouse aphid populations. To test this, we compared the performance of aphid lines originating from different greenhouses in parasitism assays with the most used parasitoids for aphid biocontrol.

#### 2.3.1 Aphid lines

Nine living lines of *M*. *persicae* and six living lines of the foxglove aphid *Aulacorthum solani* (Kaltenbach) were collected during the second sampling time point in May/June/July from both organic and conventional greenhouses (Table S1). Each line was collected from a different greenhouse, and the lines were established from a single parthenogenetic individual. We established that the lines were genetically variable from each other with the use of microsatellite analysis as part of a larger and ongoing study. Moreover, all lines were shown to be endosymbiont‐free with diagnostic PCR, following the methods described above. The aphid lines were maintained on 22.3‐mm‐diameter sweet pepper leaf discs placed on 1% agarose in 12‐well plates (665180; Greiner Bio‐One GmbH, Frickenhausen, Germany), and stored upside down at 15°C at 16:8‐hr light–dark and 60% relative humidity. A few weeks before the onset of experiments, populations were boosted by placing the aphids on larger 91‐mm‐diameter leaf discs on 1% agarose in sterile polypropylene culture vessels (Lab Associates, Oudenbosch, the Netherlands) with nylon‐screened Donut Lids (BDC0001‐1; Bugdorm, MegaView Science, Taichung, Taiwan) at 20°C.

#### 2.3.2 Parasitization tests

The nine lines of *M*. *persicae* were tested for varying levels of endogenous resistance against the parasitoids *A*. *colemani* and *A*. *matricariae*, and the six lines of *A*. *solani* were tested for resistance against the parasitoid *A*. *ervi*. Parasitoids were provided by Koppert Biological Systems (Berkel en Rodenrijs, Netherlands) as aphid mummies, and emerged wasps were kept in a nylon mesh rearing cage (4M1515, Bugdorm) and fed with a 20% honey solution. Instead of using parasitization assays where a parasitoid wasp is left with several aphids for a certain amount of time (Henter & Via, 1995; Vorburger et al., 2009), and/or where different parasitoid individuals were used for each aphid line (Martinez et al., 2014), we opted for an approach where we could observe one parasitoid parasitizing a single aphid, and where one parasitoid would subsequently parasitize an aphid of each line to minimize the effect of wasp virulence differences without the need for large wasp and aphid sample sizes. A single female wasp of at least three days old was placed in a glass vial with a second instar aphid nymph. We observed the wasp until it inserted the ovipositor, after which we replaced the nymph with another one from a different line. Each wasp was allowed to parasitize one nymph of each line. The order in which the lines were presented was randomized, and if a wasp would not parasitize within 10 min, it was discarded and a new wasp was taken. After parasitization, the aphid nymph was put on a sweet pepper leaf disc on 1% agarose in a 12‐well plate. All aphids parasitized by the same wasp were put in the same 12‐well plate in the order they were parasitized. The 12‐well plates were kept upside down in an incubator at 20°C and 60% relative humidity. We performed 70 replications per combination of aphid line and parasitoid species. All experiments were performed at 20°C, and parasitized aphids were kept in an incubator at 20°C at 16:8‐hr light–dark and 60% relative humidity.

After two weeks, the outcome of each parasitization event was scored into one of four categories: (1) the aphid survived, (2) the aphid died, (3) a mummy was formed, which did not emerge after an additional 14 days, or (4) a parasitoid wasp emerged. The sex of all emerged wasps was noted. A small number of samples failed due to the aphid being damaged during transfer, and these samples were excluded from further analyses. Parasitization rates were calculated as the ratio of mummies, both eclosed and uneclosed, to (1) the total number of replicates, and (2) the total number of replicates excluding the dead aphids (henceforth called ‘parasitism success type 1’ and ‘parasitism success type 2’, respectively). We also determined host‐death rates in two different ways: first, the rates of dead aphids to all replicates; and second, the rate of dead aphids and uneclosed mummies to all replicates (henceforth called ‘host‐death rate type 1’ and ‘host‐death rate type 2’). Proportions of successful parasitism and host‐death rates were compared between the lines.

### 2.4 Statistical analyses

All statistical analyses were performed in R version 4.0.2 (R Core Team, 2020) using RStudio version 1.3.959 (RStudio Team, 2020) unless otherwise specified.

#### 2.4.1 Aphid communities, pest management and perceived biocontrol success by growers

We tested for a significant difference between conventional and organic greenhouses in perceived ease of biocontrol using a chi‐squared test. Differences in the distribution of aphids throughout the greenhouses were tested using generalized linear mixed models (GLMMs) based on a binominal distribution with a logit‐link function, using the package *lme4* (Bates et al., 2015). We tested for the effects of greenhouse management type (organic vs. conventional) and the size of sampling site (greenhouse size in m^2^ divided by the number of cells the greenhouse was split up into). To prevent pseudoreplication, greenhouse identifier was included as a random factor. We tested the performance of the final models by assessing the normality of residuals with the *DHARMa* package (Hartig, 2021). The models were fitted for all possible factor combinations, and the best‐fitted model was chosen by comparing Akaike's information criterion (AIC) values between models. *p* values were estimated with a Wald chi‐squared test by using the ‘Anova’ function of the *car* package (Fox & Weisberg, 2019), and *p* values of the best‐fitting models were adjusted using the false discovery rate (FDR) method to correct for multiple testing (Benjamini & Hochberg, 1995).

#### 2.4.2 Detection of bacterial endosymbionts

All analyses regarding the ‘complete microbiome analysis’ are described in the respective paragraph above.

#### 2.4.3 Screening for endogenous resistance

To test whether parasitism success, host‐death rates and the sex of the emerged parasitoids differed between aphid lines, both for *M*. *persicae* and for *A*. *solani*, GLMMs based on a binomial distribution with a logit‐link function were used (*lme4* package; Bates et al., 2015). We tested the performance of the final models by assessing the normality of residuals with the *DHARMa* package (Hartig, 2021). In the case of overdispersion of variance, negative binomial models were used (*glmmTMB* package; Brooks et al., 2017). We tested for the effect of aphid line, and the parasitoid individual used for parasitizing the aphids was included as a random factor. *p* values were estimated with a Wald chi‐squared test by using the ‘Anova’ function of the *car* package (Fox & Weisberg, 2019), and adjusted using the FDR method to correct for multiple testing (Benjamini & Hochberg, 1995).

Differences in parasitism success between the two parasitoid species tested on *M*. *persicae* were also tested using GLMMs, using the same methods as described above.

## 3 RESULTS

### 3.1 Aphid communities, pest management and biocontrol success as perceived by growers

#### 3.1.1 Aphid communities

We sampled 24 greenhouse compartments belonging to 18 greenhouses during the first (February/March) sampling period, and 19 compartments belonging to 15 greenhouses during the second (May/June/July) and third (September/October) sampling periods. Different compartments located in the same greenhouse did not always contain the same aphid species and densities.

During the first sampling period, we found *M*. *persicae* in 79%, and *A*. *solani* in 29% of the visited greenhouse compartments. During the second time point, these numbers had risen to 95% and 79%, respectively, and to 100% and 89% in period three (Figure 2a). *M*. *persicae* and *A*. *solani* were by far the most common aphid species encountered in any of the greenhouses. Three other species were sampled: *Macrosiphum euphorbiae* (Thomas) and *Aphis nasturtii* Kaltenbach were encountered twice, and *A*. *fabae*, once (Figure 2a). These aphids were always found in low densities on a small number of plants.

To compare the distribution of aphids through a greenhouse, we tested for the effects of greenhouse management type and size of the sampling site on the presence of aphids at a sampling site with GLMMs. For *M*. *persicae*, the best‐performing GLMM included only greenhouse management as a fixed factor (see Table S4 for performance comparison of the different models). This effect was significant (GLMM: χ²(1, *N* = 1989) = 22.59, *p* < .001). On average, we found *M*. *persicae* in 59 ± 32% (mean ± SD) of the 36 sampling sites in organic greenhouses and in 20 ± 19% (mean ± SD) of the 36 sampling sites in conventional greenhouses (Figure 2c). Also for *A*. *solani*, only greenhouse management was included in the GLMM. Here, management did not result in a significant effect (GLMM: χ²(1, *N* = 1989) = 2.14, *p* = .143). *A*. *solani* was found in 44 ± 31% (mean ± SD) of the 36 sampling sites in organic greenhouses and in 33 ± 29% (mean ± SD) of the 36 sampling sites in conventional greenhouses (Figure 2c).

#### 3.1.2 Interviews with greenhouse growers

The growers of all greenhouses sampled in this study actively controlled aphid populations. All conventional greenhouse growers combine biocontrol agents and pesticides to control aphid pests. The pesticides used most often were pymetrozine and pirimicarb, while one grower used abamectin and one other sulfoxaflor (Table S1). All conventional and organic growers used parasitoid wasps to control aphids at the start of the growing season. *Aphidius colemani* and *A*. *ervi* were used most often (in 24 and 12 of 25 greenhouses, respectively) (Table S1). Organic growers used more frequent releases, with most growers releasing their parasitoids once or twice a week. The conventional growers released their parasitoids either once a week or once every two weeks, and often used parasitoids only in some parts of their greenhouse. Organic growers continued releasing parasitoids for most of the growing season, while 56% of conventional growers had stopped releasing parasitoids between our second and third sampling time point (Table S1). At this point, the pest management strategy differed between greenhouses, with some starting with pesticides in early spring and others waiting until the middle of summer. In addition to parasitoids, 80% of the growers also released predatory larvae of the gall midge *Aphidoletes aphidimyza* (Rondani) and 16% released coccinellid predators as well (Table S1).

Growers from organic greenhouses deemed their aphid populations significantly more often easy to control with biocontrol agents than the conventional growers (84% vs 15% considered aphid control ‘easy’, respectively, χ*^2^* (1, *N* = 45) = 21.2545, *p* < .001; Figure 2d).

### 3.2 Detection of bacterial endosymbionts

#### 3.2.1 Diagnostic PCRs

We screened 780 *M*. *persicae*, 526 *A*. *solani*, three *M*. *euphorbiae*, two *A*. *nasturtii* and one *A*. *fabae* samples for the presence of secondary endosymbiont with diagnostic PCRs. We never detected secondary endosymbionts in any of the *M*. *persicae* and *A*. *solani* individuals. However, *M*. *euphorbiae* carried *R*. *insecticola* and *A*. *nasturtii* carried *Arsenophonus* sp. Additionally, the primers targeting *S*. *symbiotica* picked up a bacterium belonging to the genus *Providencia* in *A*. *fabae*. Although *Providencia* has been detected in aphids previously (McLean et al., 2019; Moran et al., 2005), the potential function or pathogenicity of this bacterium to aphids is unknown.

#### 3.2.2 Complete microbiome analysis

To ensure that no secondary endosymbionts were missed with diagnostic PCR, the complete microbiomes of 287 out of the 1306 collected *M*. *persicae* and *A*. *solani* aphid colonies were analysed by 16S rRNA sequencing using the MinION nanopore sequencer.

We obtained between 13,785 and 26,257 raw fastq reads per sample (Table S3). After filtering the raw sequence reads for quality and size, between 8,791 and 15,447 reads per sample were left. Of the total 1,033,123 filtered reads (positive controls excluded), 99.85% were taxonomically classified to the genus level. Before subsequent cleaning of the data set by filtering out spurious taxa, 98.5% of all classified reads were assigned to *Buchnera*. After filtering out expected contaminants and rare taxa, most samples only contained *Buchnera*. Seven samples contained one or more of the following bacterial genera: *Bacillus*, *Cutibacterium*, *Enterococcus*, *Escherichia*, *Klebsiella*, *Listeria*, *Moraxella*, *Pseudomonas*, *Salmonella*, *Streptococcus*, *Synechococcus* and *Tatumella* (Figure S1). However, again, no secondary endosymbionts were observed in any of the samples, confirming our results of the diagnostic PCR for *M*. *persicae* and *A*. *solani*.

### 3.3 Screening for endogenous resistance

**FIGURE 3:**
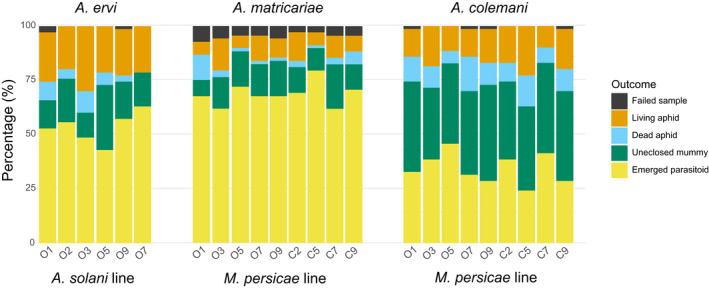
Proportions of parasitism outcome of three parasitoid wasp species on *Myzus persicae* and *Aulacorthum solani* in laboratory parasitism assays using *Aphidius ervi* on *A*. *solani* lines, and *Aphidius colemani* and *Aphidius matricariae* on *M*. *persicae* lines. For *A*. *ervi* and *A*. *matricariae*, *N* = 70, and for *A*. *colemani*, *N* = 69 per line. The lines are named after their greenhouse of origin, with the O or C representing organic and conventional. The replicates are split per aphid line and coloured by outcome. The failed samples indicate replicates where the outcome could not be clearly scored in one of the other four categories. No significant differences in parasitism success were found between the lines or pest control strategies

To assess variation in endogenous resistance, we tested nine *M*. *persicae* and six *A*. *solani* lines from different greenhouses for resistance against their most used parasitoids. We tested *M*. *persicae* against *A*. *matricariae* and *A*. *colemani*, and *A*. *solani* against *A*. *ervi*, and observed parasitism rates of approximately 83%, 74% and 72%, respectively, for parasitism rate type 1, for the total number of replicates and approximately 85%, 82% and 76%, respectively, for parasitism rate type 2, for the total number of replicates excluding the dead aphids. No significant effect of aphid line on parasitism rate type 1 and type 2, and host‐death rate type 1 and 2 was found (Figure 3; Table S5). In *M*. *persicae*, parasitism success type 1 significantly differed between *A*. *matricariae* and *A*. *colemani* (GLMM: χ*^2^* (1, *N* = 1242) = 7.36, *p* = .008), while it did not for parasitism success type 2 (GLMM: χ*^2^* (1, *N* = 1155) = 1.17, *p* = .279). Host‐death rates also differed between the two parasitoid species (type 1, GLMM: χ*^2^* (1, *N* = 1242) = 14.9, *p* < .001; type 2, GLMM: χ*^2^* (1, *N* = 1242) = 79.85, *p* < .001). Furthermore, while in *M*. *persicae* parasitism by both wasp species resulted in mummies approximately 70%–80% of the time, *A*. *matricariae* mummies successfully developed into an adult wasp significantly more often than mummies from *A*. *colemani* (GLMM: χ*^2^* (1, *N* = 967) = 70.28, *p* < .001), with 83 ± 4.6% (mean ± SD) vs 47 ± 6.4% (mean ± SD) of mummies eclosing. Another difference was observed in the sex ratios of emerging parasitoids: *A*. *colemani* had a sex ratio of 0.26 males to females, while the sex ratio of *A*. *matricariae* was 0.64 males to females (GLMM: χ*^2^* (1, *N* = 462) = 22.13, *p* < .001). The sex ratio of *A*. *ervi* on *A*. *solani* was 0.72 males to females.

## 4 DISCUSSION

Parasitoid‐based aphid biocontrol is a sustainable alternative to conventional chemical pest control strategies. However, it is expected that in protected environments such as greenhouses, high parasitoid pressure could lead to rapid selection for parasitoid‐resistant aphid lines, and consequently hampering the success of biocontrol practices (Vorburger, 2018). This will especially be the case when aphid populations can remain in the greenhouse during subsequent years, resulting in an increase in the frequency of resistant aphids over time through positive selection. This resistance could be endogenous but could also be caused by heritable bacterial endosymbionts. Variation in levels and/or presence of these heritable elements is necessary for evolution of parasitoid‐resistant aphids to proceed through natural selection. However, studies on the presence of heritable elements in greenhouse aphids are scarce and the effect of these elements on the biological control of greenhouse aphids has not been studied before. Therefore, we started by determining whether secondary endosymbionts and/or varying levels of endogenous resistance against parasitoids can be observed in greenhouse aphids.

We did not find secondary endosymbionts in the two most common aphid species, nor did we find any aphid lines with increased endogenous‐based resistance against parasitoids used for biocontrol. Because no secondary endosymbionts were detected, the objective to follow the distribution of endosymbiont‐infected aphid lines throughout the greenhouse over time became obsolete. Consequently, it was also not possible to correlate the presence of specific endosymbiont‐infected aphid lines to biocontrol success as perceived by growers. However, we did observe that aphid populations are more evenly distributed throughout organic greenhouses than conventional greenhouses (Figure 2c).

### 4.1 Aphid communities and biocontrol success as perceived by growers

The most abundant aphid species in the sampled Dutch sweet pepper greenhouses were *M*. *persicae* and *A*. *solani*. Organic growers deemed aphid populations in their crop easy to control with biocontrol agents more often than conventional growers did. When comparing aphid distributions between organic and conventional greenhouses, clear differences were observed as aphids were more evenly distributed in organic greenhouses (Figure 2c). While sampling, we also observed that aphid densities per plant were much higher in conventional greenhouses than in organic greenhouses (personal observations). We expect that this, combined with the more frequent releases of parasitoids in organic greenhouses, could explain why only 15% of the conventional growers considered it easy to control aphids with biocontrol agents compared with 84% of the organic growers. Additionally, the application of chemical pesticides in conventional greenhouses can disrupt the balance of biocontrol by untargeted effects on biocontrol agents, making biocontrol less effective (Desneux et al., 2007). Moreover, a recent synthesis using model simulations and a meta‐analysis showed that pesticide usage in a system where effective natural enemies are present can even lead to an increase in average pest densities (Janssen & van Rijn, 2021). We hypothesize that in organic sweet pepper greenhouses in the Netherlands, the presence of sufficient hosts for parasitoids, plus the frequent augmentative release of additional parasitoids, leads to a ‘standing army’ of parasitoids (Messelink et al., 2014), which can more easily respond to, and successfully suppress, growing aphid populations. We assume that in conventional greenhouses, the occasional total removal of aphids by insecticide usage results in host numbers too low for the parasitoids to build up such a ‘standing army’, making the system incapable of quickly suppressing occasional aphid outbreaks.

However, there are also downsides to the continued aphid and parasitoid presence in a greenhouse. Perhaps the most important downside is that hyperparasitoid populations can also establish more easily. Hyperparasitoids are commonly present in organic sweet pepper greenhouses in the Netherlands (Bloemhard et al., 2014), and it has been shown that they detrimentally affect parasitoid populations and consequently parasitoid‐based biocontrol success (Schooler et al., 2011; Sullivan & Völkl, 1999; Nagasaka et al., 2010). Another downside of the constant presence of aphids in an organic greenhouse is that honeydew can contaminate the product, necessitating a washing procedure (Bloemhard & Ramakers, 2008) that increases resource use, affects production time and increases product prices. Consequently, there are trade‐offs between building a strong standing army of parasitoids by offering enough hosts, and the increased risk of invading hyperparasitoids and decreased cost‐effectiveness of the crop production.

In the year of sampling, none of the sampled organic growers reported problematic aphid outbreaks. However, occasionally, sweet pepper growers experience severe yield losses due to aphid outbreaks (Bloemhard & Ramakers, 2008; Gillespie et al., 2009). During the interviews, some growers even reported to have had years where their entire crop was discarded due to uncontrollable aphid populations (personal communication). However, even though only 15% of the conventional growers found it easy to control the aphid pests in a biological manner, none of the interviewed organic sweet pepper growers experienced substantial problems to control their aphid pest populations in 2019. Consequently, it is still possible that protective endosymbionts, although not found in this study, cause uncontrollable aphid outbreaks in other years or greenhouses. To start testing this hypothesis, sampling aphids to determine possible endosymbiont presence would need to be done during highly problematic aphid outbreaks. Such a sampling scheme could be performed by biocontrol consultants, who, over prolonged periods of time, closely monitor the performance of biocontrol agents in the greenhouse.

### 4.2 Endosymbionts

We never detected secondary endosymbionts in the two main aphid species, *M*. *persicae* and *A*. *solani*, in Dutch sweet pepper greenhouses. Because we used both diagnostic PCR on 1312 aphid colonies, and whole microbiome sequencing using universal primers on 287 of these samples, we are confident that if any currently known secondary endosymbionts would have been present in these greenhouses, we would have detected them. In other studies on these aphid species, secondary endosymbionts are also rarely or never detected (Gallo‐Franco et al., 2019; Henry et al., 2015; Postic et al., 2020; Singh et al., 2021). However, since the aphid species that dominate pepper crops are likely different in other countries, our results cannot be automatically extrapolated to sweet pepper crops around the world. Other commonly dominating aphid species on sweet pepper are, for example, *M*. *euphorbiae* in Spain (Sanchez et al., 2010), and *Aphis gossypii* Glover in Colombia (Gallo‐Franco et al., 2019). Both *M*. *euphorbiae* and *A*. *gossypii* are often found carrying secondary endosymbionts (Henry et al., 2015; Najar‐Rodríguez et al., 2009). Therefore, we cannot rule out that endosymbionts affect parasitoid‐based biocontrol of aphids in protected pepper crops in other countries.

What factors determine whether and when aphids carry secondary endosymbionts is an important question. Key factors known to be correlated with endosymbiont infection are host plant genus (Henry et al., 2015), plant diversity (Zytynska et al., 2016), temperature (Doremus et al., 2018; Smith et al., 2021) and geographic location (Zytynska & Weisser, 2016). *M*. *persicae* and *A*. *solani* are two of the most economically important and problematic pest species in many crops worldwide (Jandricic et al., 2010; van Emden & Harrington, 2017). Our results add to the evidence that secondary endosymbionts rarely occur in these aphid species (Gallo‐Franco et al., 2019; Henry et al., 2015; Postic et al., 2020). Hence, it is unlikely that secondary endosymbionts play an important role in protecting these aphid species against parasitoids used for biocontrol. Possibly, other protection mechanisms play a role in protecting these aphid species against their hymenopteran enemies.

One such, currently unexplored, mechanism might be the horizontal transfer of prokaryotic genes involved in parasitoid resistance into the genomes of some aphid species. An example of this was presented by Verster et al. (2019), who reported horizontal transfer of the eukaryotic genotoxin cytolethal distending toxin B (CdtB) gene into the genome of some aphid species, among which, *M*. *persicae*. CdtB is also encoded by APSE‐2, APSE‐6 and APSE‐7 bacteriophages of *H*. *defensa* (Rouïl et al., 2020). Therefore, the presence of CdtB in the genome of *M*. *persicae* could obviate the need for this aphid to carry protective endosymbionts, especially when there are endosymbiont‐related costs to the aphid. Functional studies to test whether aphid‐encoded CdtB plays a role in protection against parasitoids would be a logical next step.

### 4.3 Endogenous resistance

Aside from protection by endosymbionts, endogenous‐based resistance against parasitoids is also observed in aphids. We hypothesized that high parasitoid pressure in organic greenhouses might select for aphids with high endogenous resistance. However, no variation in levels of endogenous resistance against common biocontrol parasitoids was observed between lines of both *M*. *persicae* and *A*. *solani* from different greenhouses (Figure 3). Without variation in levels of resistance, it is unlikely that selection can lead to highly resistant aphid populations. However, there is ample evidence for endogenous‐based resistance in pea aphids (Doremus et al., 2018; Martinez et al., 2014, 2016; McLean & Parker, 2020) and clonal variation in resistance against *A*. *colemani* has been observed before as well in *M*. *persicae* (von Burg et al., 2008). Additionally, *M*. *persicae* is a species well known for its ability to develop resistance against insecticides (Bass et al., 2014), and insecticide resistance has been linked to increased susceptibility to parasitoids (Foster et al., 2007). The fact that we did not find signatures for endogenous resistance might be caused by our experimental setup. Since we made sure that the parasitoids had stung the aphids, our methods tested only for physiological resistance mechanisms, and not for differences between aphid lines in parasitoid search behaviour and host acceptance. It is known that aphid size, shape, colour, odour, induced plant volatiles and aphid defence tactics are all factors that can affect parasitoid preference for a host (Rehman & Powell, 2010). These factors could still differ in our tested lines and affect the success of parasitoids on a greenhouse scale.

It is also possible that we did not find variation in resistance against parasitoids because aphids are parasitized and predated upon by multiple species in a greenhouse setting. In a simplistic two‐species parasitoid–host relationship, parasitoid–host coevolution is expected to occur, with aphids evolving increased resistance and parasitoids evolving increased virulence. However, these two‐species interactions do not accurately represent real‐life situations in greenhouses where often multiple parasitoid genotypes and/or species are present, in addition to multiple predators. It is common that natural enemies that were never deployed by the grower establish themselves in a greenhouse (e.g. Bosco et al., 2008; Gavkare et al., 2014; Postic et al., 2021). It has been shown that the presence of both specialist and generalist parasitoids can stabilize biological control (Raymond et al., 2016). The presence of multiple parasitoid genera, species and lines will increase the chance that aphid resistance can be circumvented (Cayetano & Vorburger, 2015; McLean & Godfray, 2015; Rouchet & Vorburger, 2012), and parasitoid‐resistant aphids can still be preyed upon. It is worth investigating the potential role of multispecies networks in mitigating effects of endosymbionts and/or endogenous resistance on biocontrol success.

## 5 CONCLUSIONS

To summarize, we found significant differences in perceived biocontrol success between organic and conventional growers. Moreover, we found no evidence for variation in heritable resistance elements against parasitoids in aphid lines, neither caused by protective endosymbionts nor caused by endogenous‐based resistance, in sweet pepper greenhouses in the Netherlands in 2019. However, our findings do not exclude a role of these heritable elements in limiting (the evolution of) parasitoid success in other greenhouse crops, or in other aphid species. To achieve a complete picture of the role of endosymbionts and endogenous resistance in biocontrol efficacy, multiple crop systems, climates and geographic locations will need to be monitored. Obtaining this information is vital to reduce our dependency on chemical pesticides that are detrimental to the environment, and to enable a sustainable and safe control of these important greenhouse pests.

## CONFLICT OF INTEREST

The authors declare that they have no conflict of interest.

## Supporting information

Appendix S1

Table S1‐S5

## Data Availability

Data for this study and R scripts used for analyses are available on figshare and are accessible via the following link: https://figshare.com/projects/Greenhouse_aphids_lack_heritable_protection/130715. Raw sequence data have been deposited in the Sequence Read Archive (http://www.ncbi.nlm.nih.gov/sra) under the BioProject number PRJNA801219.
