# Supplementary material for: Do aphids in Dutch sweet pepper greenhouses carry heritable elements that protect them against biocontrol parasitoids?
Source: Evol Appl. 2022 Feb 15;15(10):1580–93. doi: 10.1111/eva.13347 (PMC9624084; doi:10.1111/eva.13347)
Supplement: Supplementary file 1 — Appendix S1 [file EVA-15-1580-s002.pdf]

Supplementary materials for

Do aphids in Dutch sweet pepper greenhouses carry heritable elements that protect them against biocontrol parasitoids?

Mariska M. Beekman, S. Helena Donner, Jordy J. H. Litjens, Marcel Dicke, Bas J. Zwaan , Eveline C. Verhulst, and Bart A. Pannebakker

Correspondence to: mariska.beekman@wur.nl
ORCID https://orcid.org/0000-0003-0498-5487

This PDF file includes:

Supplementary Text S1: Methods for determining cutoff values for filtering of wrongly assigned taxa after 16S rRNA sequencing

Supplementary Text S2: Pilot experiment pooling aphid DNA samples for endosymbiont detection

Supplementary Figure S1: Rarefied abundance of bacterial genera detected in aphids with nanopore 16S rRNA sequencing.

Text S1: Methods for determining cutoff values for filtering of wrongly assigned taxa after 16S rRNA sequencing

Taxa and reads assigned to the ZymoBIOMICS Microbial Community Standard (D6300), and the ZymoBIOMICS Microbial DNA Standard (D6305), were used to determine the cut-off values for filtering out wrongly assigned taxa due to sequencing artifacts, sequencing errors, and incorrectly assigned barcodes.

Of the total 1016178 reads assigned to *Buchnera*, approximately 0.5% contained the barcodes belonging to the ZymoBIOMICS standards samples. It is expected that these reads were assigned to the wrong barcode, probably due to the wrong barcode attaching to the reads during barcoding PCR. This rough estimation of reads having a wrong barcode is similar to the 0.3% that was observed by Wick, Judd, & Holt (2018). This wrong assignment of barcodes probably affected only reads assigned to *Buchnera*, since these made up 98.5% of the total reads. For the second most abundant genus in our sequencing setup, *Staphylococcus*, this would already come down to less than one wrongly assigned read per sample.

Remarkable was that 10.2 and 5.5% of the total reads of the ZymoBIOMICS Microbial Community Standard and the ZymoBIOMICS Microbial Community DNA Standard respectively, were assigned to *Klebsiella*, which is not part of the original composition of these samples. Since *Klebsiella* was detected in only one aphid sample, we exclude the possibility of contamination. Genera belonging to the Enterobacteriaceae family have high sequence similarity in the 16S rRNA gene, making distinguishing between genera difficult for classifiers (Wood & Salzburg, 2014). Since *Salmonella* belongs to the Enterobacteriaceae and thus has a 16S rRNA sequence highly similar to that of *Klebsiella*, we expect that this, in combination with the high error rate of MinION sequencing and the k-mer based identification approach, can explain the observed high percentages of reads assigned to *Klebsiella.*

Text S2: Pilot experiment pooling aphid DNA samples for endosymbiont detection

Aim of experiment

Determine the sensitivity of endosymbiont diagnostic PCR analysis of aphid-extracted DNA. For this we diluted endosymbiont-infected aphid DNA with uninfected-aphid DNA to try to still detect the endosymbiont in diagnostic PCR. In other words, we tried to determine how much aphid DNA can be pooled while still being able to pick up endosymbionts present in a single aphid.

Methods

Aphids samples

Samples were kindly send to us by Christoph Vorburger in DNA-grade EtOH.
U: A5 (*Acyrthosiphon pisum* uninfected)
R: 5A-U (*Acyrthosiphon pisum* with Regiella insecticola)
H: 5A-T (*Acyrthosiphon pisum* with Hamiltonella defensa)
S: C161 (*Acyrthosiphon pisum* with Spiroplasma sp.)

DNA extraction and PCR

DNA was extracted with a Chelex + proteinase K protocol as described in the main manuscript. Multiplex PCRs were mostly performed as described in the main manuscript, with the only modification being the primer used to *detect Buchnera aphidicola* (see Suppl. Table S2 for primer details)

DNA samples in PCR
Samples were made up by mixing DNA of samples U, R, H and S in different ratios and using 1uL of the DNA mix as the input material for PCR amplification. See Table A for the composition of the samples.

***Table A**: creation of DNA samples to test sensitivity of diagnostic PCR*

	parts				
	U (uninfected)	R (*Regiella insecticola*)	H (*Hamiltonella defensa*)	S (Spiroplasma)	Dilution
1	-	1	1	1	3x
2	4	1	-	-	5x
3	4	-	1	-	5x
4	4	-	-	1	5x
5	9	1	-	-	10x
6	9	-	1	-	10x
7	9	-	-	1	10x
8	9	1/3	1/3	1/3	30x
9	19	1	-	-	20x
10	19	-	1	-	20x
11	19	-	-	1	20x
12	19	1/3	1/3	1/3	60x

Analysis

Amplicons were visualized by gel electrophoresis on a 2% agarose gel stained with EtBr with the GeneRuler 100bp DNA ladder. Amplicons of the expected size (see Suppl. Table S2) were cut from the gel and sequences were confirmed by Sanger sequencing.

Results

With all samples a bright band was observed for *Buchnera*. Both *Hamiltonella* and *Regiella* were clearly observable in all dilutions, both in samples where they were the only endosymbiont present (5x, 10x, 20x) as well as in samples where all three endosymbionts were present (3x, 30x, 60x) (see Figure A).

For *Spirolasma*, the bands observed were much fainter than for *Hamiltonella* and *Regiella*. However, when it was the only endosymbiont present it could still be detected at 20x dilution. In a mix with the other endosymbionts, a faint smear in the correct size range was detected at 3x dilution, a band was barely detectable at 30x dilution and not detectible at 60x dilution. For both *Regiella* and *Spiroplasma*, besides amplicons of the expected size, also some faint bands of other lengths were observed.

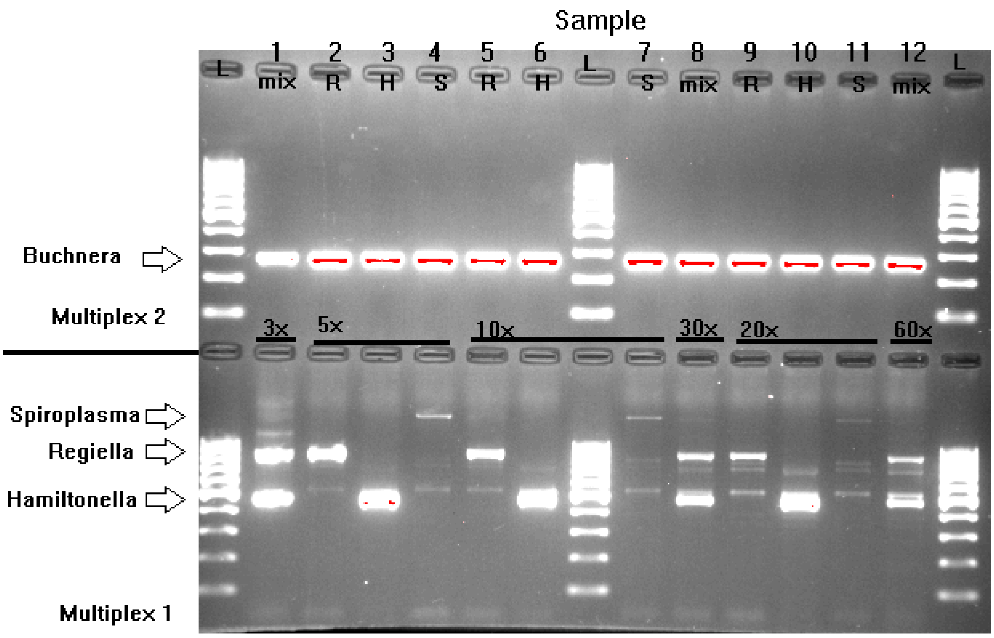

Figure A: *PCR results visualized on a 2% agarose gel stained with EtBr.* L: GeneRuler 100bp DNA ladder. Mix: DNA mix containing DNA from the aphids containing *Regiella insecticola*, *Hamiltonella defensa* and *Spiroplasma*. R: *R. insecticola* only. H: *H. defensa* only. S: *Spiroplasma* only. 3x, 5x, 10x, 30x, 20x and 60x show how much the original (single symbiont containing) DNA was diluted.

Discussion & Conclusions

DNA from endosymbiont-infected aphids for which a bright band is obtained in singleplex can be easily diluted and will still result in detection of endosymbionts, even when DNA is diluted up to 20x with DNA from uninfected aphids and up to 60x when DNA from multiple aphids carrying different endosymbionts are present. For endosymbionts for which a more faint band is obtained in singleplex, the detection threshold is a little lower. When it is the only endosymbiont present in a sample, it can still be detected at at least 20x dilution (as this was our highest dilution for single symbiont samples). In a mix of DNA we were able to detect the symbiont up to 30x dilution.

For endosymbionts for which a larger PCR product is expected, the relative signal will decrease when in a mix with endosymbionts for which a smaller PCR product is expected as smaller amplicons will amplify more efficiently during PCR than larger amplicons. When endosymbionts with smaller amplicon size are detected in a mixed sample, it might be possible that endosymbionts with large amplicon sizes are missed. Therefore, in pooled samples testing positive for a small amplicon symbiont, it might be necessary to also test all primers for large amplicon symbionts in singleplex to make sure no symbionts are missed. However, our results show that a negative result of a mixed DNA sample (at least up to 20x) can be assumed to be truly negative.

Furthermore, some extra bands of unexpected lengths were observed. Therefore, we would recommend to always confirm suspected positive samples by singleplex PCR and Sanger sequencing.

References

Fukatsu, T., & Nikoh, N. (1998). Two intracellular symbiotic bacteria from the mulberry psyllid Anomoneura mori (insecta Homoptera). Applied and Environmental Microbiology, 64(10), 3599–3606. https://doi.org/10.1128/aem.64.10.3599-3606.1998

Guay, J.-F., Boudreault, S., Michaud, D., & Cloutier, C. (2009). Impact of environmental stress on aphid clonal resistance to parasitoids: Role of Hamiltonella defensa bacterial symbiosis in association with a new facultative symbiont of the pea aphid. Journal of Insect Physiology, 55(10), 919–926. https://doi.org/10.1016/J.JINSPHYS.2009.06.006

McLean, A. H. C., Van Asch, M., Ferrari, J., & Godfray, H. C. J. (2011). Effects of bacterial secondary symbionts on host plant use in pea aphids. Proceedings of the Royal Society B: Biological Sciences, 278(1706), 760–766. https://doi.org/10.1098/rspb.2010.1654

Peccoud, J., Bonhomme, J., Mahéo, F., de la Huerta, M., Cosson, O., & Simon, J. C. (2014). Inheritance patterns of secondary symbionts during sexual reproduction of pea aphid biotypes. Insect Science, 21(3), 291–300. https://doi.org/10.1126/science.1195463.

Tsuchida, T., Koga, R., Horikawa, M., Tsunoda, T., Maoka, T., Matsumoto, S., … Fukatsu, T. (2010). Symbiotic Bacterium Modifies Aphid Body Color. Science, 330(6007), 1102–1104. https://doi.org/10.1126/science.1195463

Wick, R. R., Judd, L. M., & Holt, K. E. (2018). Deepbinner: Demultiplexing barcoded Oxford Nanopore reads with deep convolutional neural networks. *PLoS Computational Biology*, *14*(11), e1006583. https://doi.org/10.1371/journal.pcbi.1006583

Wood, D. E., & Salzberg, S. L. (2014). Kraken: Ultrafast metagenomic sequence classification using exact alignments. *Genome Biology*, *15*(3). https://doi.org/10.1186/gb-2014-15-3-r46

Supplementary Figure S1

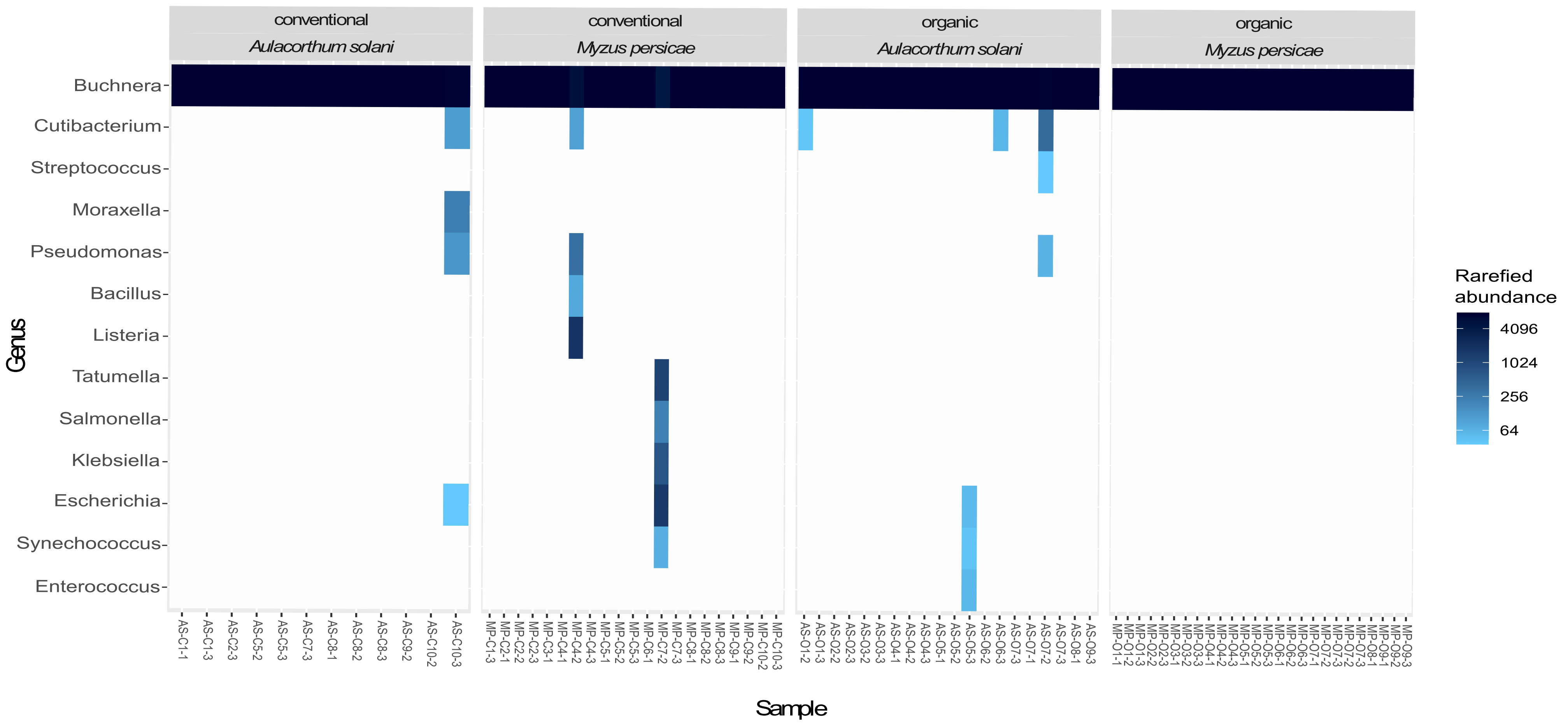

Figure S1: Rarefied abundance of bacterial genera detected in aphids with nanopore 16S rRNA sequencing. Heatmap showing the abundance (rarefied number of reads) of bacterial genera, determined by 16S rRNA sequencing, in aphid samples from sweet pepper greenhouses. Samples are grouped by aphid species and pest control strategy of the respective greenhouse where aphids were sampled. Samples are named according to the aphid species (MP = *Myzus persicae*, AS = *Aulacorthum solani*), the identifier of the greenhouse they are collected from (see table S3), and the timepoint of sampling (1 = Feb/March, 2 = May/June/July, 3 = Sept/Oct). Samples with identical identifiers come from different compartments within the greenhouse.
